# Supplementary material for: Vitamin D Supplementation Does Not Influence SARS-CoV-2 Vaccine Efficacy or Immunogenicity: Sub-Studies Nested within the CORONAVIT Randomised Controlled Trial
Source: Nutrients. 2022 Sep 16;14(18):3821. doi: 10.3390/nu14183821 (PMC9506404; doi:10.3390/nu14183821)
Supplement: Supplementary file 1 [file nutrients-14-03821-s001.zip › nutrients-1897264-supplementary.pdf]

Vitamin D Supplementation does not influence SARS-CoV-2 Vaccine Efficacy or Immunogenicity: Sub-studies Nested within the CORONAVIT Randomised Controlled Trial.

Supplementary Appendix

Contents

Table S1. Baseline characteristics of participants contributing data to analysis of post-vaccination anti-S IgGAM titres (sub-study 2), by allocation 2

Table S2. Baseline characteristics of participants contributing data to analysis of neutralising antibody titres and cellular responses (sub-study 3), by allocation 3

Table S3: Dichotomous outcomes by allocation 4

Table S4: Continuous immunological outcomes by allocation: intervention arms pooled 5

Table S5: Sensitivity analysis: vaccine efficacy analysis in participants who did not report previous SARS-CoV-2 infection 7

Table S6: Sensitivity analysis: dichotomous outcomes by allocation in participants who did not report previous SARS-CoV-2 infection 7

Table S7: Sensitivity analysis: continuous immunological outcomes in participants who did not report previous SARS-CoV-2 infection 7

Table S8: Exploratory responder analysis, comparing major immunological outcomes among participants randomised to either intervention arm who did vs. did not achieve end-trial 25(OH)D concentrations >75 nmol/L. 8

Table S1. Baseline characteristics of participants contributing data to analysis of post-vaccination anti-S IgGAM titres (sub-study 2), by allocation

	**Overall (n=1853)**	**No offer (n=555)**	**800 IU/day offer (n=646)**	**3200 IU/day offer (n=652)**
Age, years				
Median (IQR)	63.6 (57.0–69.3)	63.5 (57.2–69.2)	63.0 (56.5–68.8)	64.1 (57.0–70.1)
Range	16.6–87.2	18.4–82.8	26.8–83.1	16.6–87.2
Sex				
Female	1,228 (66.3%)	341 (61.4%)	447 (69.2%)	440 (67.5%)
Male	625 (33.7%)	214 (38.6%)	199 (30.8%)	212 (32.5%)
Ethnicity				
White	1,805 (97.4%)	546 (98.4%)	629 (97.4%)	630 (96.6%)
Asian/Asian British	12 (0.6%)	4 (0.7%)	0	8 (1.2%)
Black/African/Caribbean/Black British	4 (0.2%)	1 (0.2%)	2 (0.3%)	1 (0.2%)
Mixed/Multiple/Other	32 (1.7%)	4 (0.7%)	15 (2.3%)	13 (2.0%)
Body-mass index, kg/m²				
<25	832 (45.0%)	238 (43.0%)	289 (44.9%)	305 (46.8%)
25–30	657 (35.5%)	208 (37.6%)	216 (33.5%)	233 (35.7%)
>30	360 (19.5%)	107 (19.3%)	139 (21.6%)	114 (17.5%)
Self-assessed general health				
Excellent	417 (22.5%)	131 (23.6%)	136 (21.1%)	150 (23.0%)
Very good	786 (42.4%)	250 (45.0%)	276 (42.7%)	260 (39.9%)
Good	469 (25.3%)	116 (20.9%)	177 (27.4%)	176 (27.0%)
Fair	143 (7.7%)	48 (8.6%)	45 (7.0%)	50 (7.7%)
Poor	37 (2.0%)	10 (1.8%)	12 (1.9%)	15 (2.3%)
Medically diagnosed disease				
Hypertension	409 (22.1%)	131 (23.6%)	137 (21.2%)	141 (21.6%)
Diabetes	96 (5.2%)	33 (5.9%)	24 (3.7%)	39 (6.0%)
Heart disease	77 (4.2%)	25 (4.5%)	26 (4.0%)	26 (4.0%)
Asthma	244 (13.2%)	66 (11.9%)	102 (15.8%)	76 (11.7%)
COPD	32 (1.7%)	12 (2.2%)	11 (1.7%)	9 (1.4%)
Previous SARS-CoV-2 infection(1)	75 (4.1%)	19 (3.5%)	35 (5.4%)	21 (3.3%)
Type of vaccine administered, primary course				
2 × ChAdOx1	1,280 (69.1%)	389 (70.1%)	463 (71.7%)	428 (65.6%)
2 × BNT162b2	559 (30.2%)	161 (29.0%)	180 (27.9%)	218 (33.4%)
Other(2)	14 (0.8%)	5 (0.9%)	3 (0.5%)	6 (0.9%)
Inter-dose interval, days	77 (70–79)	77 (70–79)	77 (70–79)	77 (70–79)
Interval from second vaccine dose to DBS sample, days	54 (41–70)	56 (41–71)	52 (41–67)	55 (41–71)
Mean 25(OH)D, nmol/L (SD) [range](3)	40.5 (14.3) [10.3–73.3]	--(4)	40.4 (14.5) [10.3–73.5]	40.7 (14.2) [10.3–73.2]
<25.0	183 (9.9%)	--(4)	96 (14.9%)	87 (13.3%)
25.0 to <50.0	766 (41.3%)	--(4)	376 (58.2%)	390 (59.8%)
50.0 to <75.0	344 (18.6%)	--(4)	172 (26.6%)	172 (26.4%)
≥75.0	0	--(4)	0	0
Not determined	560 (30.2%)	555 (100.0%)	2 (0.3%)	3 (0.5%)

Data are n (%) or median (IQR) unless specified otherwise. Abbreviations: anti-S IgGAM, combined anti-Spike IgG, IgA and IgM response; IQR, interquartile range; DBS, dried blood spot; SD, standard deviation; 25(OH)D, 25-hydroxyvitamin D. (1) Reported test-positive infection before provision of DBS sample. (2) One participant receiving 2 × Moderna (3200 IU/day offer), two participants receiving 2 × Novavax (no offer), two participants receiving 2 × Valneva (3200 IU/day offer), eight participants receiving a combination of vaccines (3200 IU/offer: n=3; 800 IU/day offer: n=3; no offer: n=2), and one participant unknown vaccine types (no offer). (3) Missing values: 25(OH)D concentration missing for three participants in 3200 IU/day arm and two participants in 800 IU/day arm. (4) Baseline 25(OH)D not determined for participants randomly assigned to the no offer arm.

Table S2. Baseline characteristics of participants contributing data to analysis of neutralising antibody titres and cellular responses (sub-study 3), by allocation

	**Overall (n=100)**	**No offer (n=31)**	**800 IU/day offer (n=29)**	**3200 IU/day offer (n=40)**
Age, years				
Median (IQR)	66.5 (61.4–68.9)	66.5 (59.7–69.5)	66.6 (64.2–69.0)	66.4 (62.0–68.3)
Range	16.6–87.2	18.4–82.8	26.8–83.1	16.6–87.2
Sex				
Female	59 (58.4%)	15 (48.4%)	19 (65.5%)	25 (62.5%)
Male	41 (41.0%)	16 (51.6%)	10 (34.5%)	15 (37.5%)
Ethnicity				
White	96 (96.0%)	30 (96.8%)	27 (93.1%)	39 (97.5%)
Asian/Asian British	1 (1.0%)	0	0	1 (2.5%)
Black/African/Caribbean/Black British	0	0	0	0
Mixed/Multiple/Other	3 (3.0%)	1 (3.2%)	2 (6.9%)	0
Body-mass index, kg/m²				
<25	45 (45.0%)	15 (48.4%)	13 (44.8%)	17 (42.5%)
25–30	44 (44.0%)	15 (48.4%)	11 (37.9%)	18 (45.0%)
>30	11 (11.0%)	1 (3.2%)	5 (17.2%)	5 (12.5%)
Self-assessed general health				
Excellent	26 (26.0%)	7 (22.6%)	7 (24%)	12 (30.8%)
Very good	39 (39.0%)	10 (32.3%)	13 (45%)	16 (41.0%)
Good	28 (28.0%)	10 (32.3%)	8 (28.0%)	10 (25.6%)
Fair	5 (5.0%)	4 (12.9%)	0	1 (2.6%)
Poor	1 (1.0%)	0	1 (3.0%)	0
Medically diagnosed disease				
Hypertension	27 (27.0%)	7 (22.6%)	9 (31.0%)	11 (27.5%)
Diabetes	8 (8.0%)	3 (9.7%)	0	5 (12.5%)
Heart disease	3 (3.0%)	1 (3.2%)	0	2 (5.0%)
Asthma	14 (14.0%)	4 (12.9%)	7 (24.1%)	3 (7.5%)
COPD	2 (2.0%)	1 (3.2%)	1 (3.4%)	0
Previous SARS-CoV-2 infection(1)	2 (2.0%)	0	0	2 (5.0%)
Type of vaccine administered, primary course				
2 × ChAdOx1	67 (67.0%)	22 (71.0%)	22 (75.9%)	23 (57.5%)
2 × BNT162b2	33 (33.0%)	9 (29.0%)	7 (24.1%)	17 (42.5%)
Other	0	0	0	0
Inter-dose interval, days	76 (70–78)	75 (70–78)	77 (70–80)	76 (70–78)
Interval from second vaccine dose to blood sample, days	82 (69–103)	82 (65–93)	81 (65–101)	83 (71–125)
Mean 25(OH)D, nmol/L (SD) [range](2)	39.3 (13.1)	--(2)	38.2 (14.1)	40.1 (12.4)
<25.0	8 (8.0%)	--(2)	4 (13.8%)	4 (10.0%)
25.0 to <50.0	48 (48.0%)	--(2)	21 (72.4%)	27 (67.5%)
50.0 to <75.0	13 (13.0%)	--(2)	4 (13.8%)	9 (22.5%)
≥75.0	0	--(2)	0	0
Not determined	31 (31.0%)	31 (100.0%)	0	0

Data are n (%) or median (IQR) unless specified otherwise. Abbreviations: IQR, interquartile range; SD, standard deviation; 25(OH)D, 25-hydroxyvitamin D. (1) Reported test-positive infection before provision of blood sample. (2) Baseline 25(OH)D not determined for participants randomly assigned to the no-offer arm.

Table S3: Dichotomous outcomes by allocation

	No offer	800 IU/day offer	3200 IU/day offer	Either offer	800 IU/day *vs* no offer		3200 IU/day *vs* no offer		Either offer *vs* no offer	
					Adjusted OR (95% CI)	P	Adjusted OR (95% CI)	P	Adjusted OR (95% CI)	P
Proportion with breakthrough SARS-CoV-2 infection, sub-study 1	49/908 (5.4%)	66/944 (7.0%)	59/956 (6.2%)	125/1900 (6.6%)	1.34 (0.91 to 1.97)*	0.141	1.19 (0.80 to 1.76)*	0.392	1.26 (0.89 to 1.77)*	0.192
Proportion of participants with detectable anti-S IgGAM post vaccination, sub-study 2	533/555 (96.0%)	628/646 (97.2%)	638/652 (97.9%)	1266/1298 (97.5%)	1.29 (0.64 to 2.60)**	0.474	1.66 (0.80 to 3.42)**	0.172	1.42 (0.78 to 2.59)**	0.248

Abbreviations: IQR, interquartile range; OR, odds ratio. *Adjusted for age, sex, educational attainment, frontline worker status, number of people per bedroom, schoolchildren (5–15 years) at home with participant, primary vaccination course, previous SARS-CoV-2 infection, season of first vaccination, inter-dose interval, and use of anticholinergics. **Adjusted for age, sex, body-mass index, days from second vaccine dose to DBS sample, general health, season of second vaccination, inter-dose interval, primary vaccination course, and previous SARS-CoV-2 infection.

Table S4: Continuous immunological outcomes by allocation: intervention arms pooled

	No offer	Either offer	Adjusted % difference for either offer vs. no offer (95% CI)*	P†
**Sub-study 2**				
Anti-S IgGAM ratio	2.8 (1.9 to 3.9) [n=555]	2.9 (1.9 to 4.1) [n=1298]	-46.6% (-85.6 to 98.0)	0.344
**Sub-study 3**				
Neutralising antibody titre	186.9 (119.8 to 406.7) [n=29]	237.6 (81.2 to 492.3) [n=66]	-2.4% (-39.5 to 57.5)	0.920
S peptide-stimulated IFN-γ in whole blood supernatant, ng/mL	0.011 (0.003 to 0.025) [n=29]	0.013 (0.005 to 0.037) [n=65]	59.1% (-22.2 to 225.3)	0.200
S peptide-stimulated TNF in whole blood supernatant, ng/mL	0.000 (0.000 to 0.003) [n=29]	0.000 (0.000 to 0.005) [n=67]	-45.1% (-78.1 to 37.5)	0.197
S peptide-stimulated IL-6 in whole blood supernatant, ng/mL	0.136 (0.012 to 0.719) [n=28]	0.125 (0.000 to 1.909) [n=66]	-48.2% (-88.1 to 125.0)	0.375
S peptide-stimulated CXCL-8 in whole blood supernatant, ng/mL	0.790 (0.254 to 2.563) [n=26]	0.909 (0.371 to 5.917) [n=66]	13.7% (-61.1 to 233.0)	0.812
LPS-stimulated IFN-γ in whole blood supernatant, ng/mL	0.020 (0.004 to 0.067) [n=29]	0.028 (0.007 to 0.072) [n=65]	29.7% (-46.5 to 214.3)	0.561
LPS-stimulated TNF in whole blood supernatant, ng/mL	0.542 (0.465 to 0.919) [n=29]	0.513 (0.319 to 0.923) [n=68]	-37.2% (-72.7 to 44.4)	0.270
LPS-stimulated IL-6 in whole blood supernatant, ng/mL	54.768 (32.362 to 73.147) [n=29]	44.478 (30.651 to 81.109) [n=68]	3.1% (-27.6 to 46.8)	0.865
LPS-stimulated CXCL-8 in whole blood supernatant, ng/mL	4.231 (2.322 to 6.373) [n=28]	2.642 (2.072 to 4.341) [n=68]	-20.2% (-44.2 to 14.2)	0.214
Percentage of S peptide-stimulated CD3+CD4+ cells positive for intracellular IFN-γ	0.00% (0.00 to 0.02) [n=29]	0.00% (0.00 to 0.02) [n=64]	-23.1% (-53.4 to 26.9)	0.299
Percentage of CD3-stimulated CD3+CD4+ cells positive for intracellular IFN-γ	0.04% (0.00 to 0.12) [n=29]	0.10% (0.02 to 0.39) [n=63]	193.8% (11.0 to 677.8)	0.030
Percentage of S peptide-stimulated CD3+CD8+ cells positive for intracellular IFN-γ	0.00% (0.00 to 0.04) [n=29]	0.00% (0.00 to 0.06) [n=64]	18.9% (-40.6 to 138.0)	0.621
Percentage of CD3-stimulated CD3+CD8+ cells positive for intracellular IFN-γ	0.62% (0.18 to 2.15) [n=29]	0.95% (0.26 to 2.09) [n=63]	76.3% (-22.4 to 300.6)	0.173
Percentage of S peptide-stimulated CD3+CD4+ cells positive for intracellular IL-2	0.00% (0.00 to 0.02) [n=29]	0.01% (0.00 to 0.02) [n=64]	26.8% (-11.7 to 81.9)	0.195
Percentage of CD3-stimulated CD3+CD4+ cells positive for intracellular IL-2	0.12% (0.09 to 0.25) [n=29]	0.18% (0.07 to 0.35) [n=63]	51.3% (-13.7 to 165.5)	0.146
Percentage of S peptide-stimulated CD3+CD8+ cells positive for intracellular IL-2	0.00% (0.00 to 0.00) [n=29]	0.00% (0.00 to 0.00) [n=64]	21.4% (-17.7 to 78.9)	0.323
Percentage of CD3-stimulated CD3+CD8+ cells positive for intracellular IL-2	0.13% (0.05 to 0.26) [n=29]	0.18% (0.08 to 0.41) [n=63]	60.6% (-13.1 to 196.8)	0.129
Percentage of S peptide-stimulated CD3+CD4+ cells positive for intracellular TNF	0.00% (0.00 to 0.00) [n=29]	0.00% (0.00 to 0.02) [n=64]	36.1% (-24.2 to 144.3)	0.297
Percentage of CD3-stimulated CD3+CD4+ cells positive for intracellular TNF	0.37% (0.19 to 0.67) [n=29]	0.73% (0.24 to 1.31) [n=63]	90.0% (-3.7 to 275.1)	0.064
Percentage of S peptide-stimulated CD3+CD8+ cells positive for intracellular TNF	0.02% (0.00 to 0.06) [n=29]	0.00% (0.00 to 0.10) [n=64]	-31.9% (-70.1 to 54.8)	0.354
Percentage of CD3-stimulated CD3+CD8+ cells positive for intracellular TNF	1.45% (0.72 to 3.84) [n=29]	2.48% (1.05 to 4.40) [n=63]	135.2% (4.9 to 427.3)	0.038
Percentage of CD3+CD4+ cells with naive phenotype	35.8% (21.7 to 48.7) [n=29]	36.5% (21.5 to 42.9) [n=64]	-3.6% (-22.4 to 19.7)	0.737
Percentage of CD3+CD8+ cells with naive phenotype	32.5% (22.9 to 42.6) [n=29]	24.6% (19.6 to 34.9) [n=64]	-13.7% (-28.9 to 4.7)	0.134
Percentage of CD3+CD4+ cells with central memory phenotype	33.5% (26.6 to 40.6) [n=29]	36.5% (30.8 to 46.1) [n=64]	18.5% (1.1 to 39.1)	0.037
Percentage of CD3+CD8+ cells with central memory phenotype	20.5% (13.9 to 28.8) [n=29]	23.0% (14.1 to 30.5) [n=64]	19.7% (-8.8 to 57.2)	0.192
Percentage of CD3+CD4+ cells with effector memory phenotype	11.7% (7.3 to 15.7) [n=29]	13.6% (9.5 to 17.8) [n=64]	13.2% (-9.5 to 41.4)	0.274

	No offer	Either offer	Adjusted % difference for either offer vs. no offer (95% CI)*	P†
Percentage of CD3+CD8+ cells with effector memory phenotype	8.2% (5.6 to 12.7) [n=29]	12.0% (7.1 to 15.1) [n=64]	34.3% (2.8 to 75.4)	0.031
Percentage of CD3+CD4+ cells with EMRA phenotype	3.9% (2.4 to 7.9) [n=29]	3.4% (2.2 to 5.8) [n=64]	-32.4% (-53.6 to -1.6)	0.041
Percentage of CD3+CD8+ cells with EMRA phenotype	14.9% (10.3 to 29.7) [n=29]	19.6% (11.2 to 27.6) [n=64]	7.8% (-21.3 to 47.7)	0.638

Data are median (IQR) [n] unless otherwise specified. Values below the limit of detection are presented as 0. *Adjusted for age, sex, ethnicity, body-mass index, days from second vaccine dose to DBS sample, general health, inter-dose interval, and primary vaccination course. †Correction for multiple testing using the Benjamin and Hochberg procedure provides a critical p value of 0.0017. EMRA, terminally differentiated effector memory cells re-expressing CD45RA.

Table S5: Sensitivity analysis: vaccine efficacy analysis in participants who did not report previous SARS-CoV-2 infection

	No offer	800 IU/day offer	3200 IU/day offer	Either offer	800 IU/day *vs* no offer		3200 IU/day *vs* no offer		Either offer *vs* no offer	
					Adjusted HR (95% CI)	P	Adjusted HR (95% CI)	P	Adjusted HR (95% CI)	P
Median days to breakthrough SARS-CoV-2 infection (IQR)	123 (100 to 145)	127 (90 to 146)	103 (58 to 139)	117 (73 to 145)	1.27 (0.88 to 1.83)	0.208	1.16 (0.80 to 1.69)	0.427	1.23 (0.89 to 1.70)	0.221

Table S6: Sensitivity analysis: dichotomous outcomes by allocation in participants who did not report previous SARS-CoV-2 infection

	No offer	800 IU/day offer	3200 IU/day offer	Either offer	800 IU/day *vs* no offer		3200 IU/day *vs* no offer		Either offer *vs* no offer	
					Adjusted OR (95% CI)	P	Adjusted OR (95% CI)	P	Adjusted OR (95% CI)	P
Proportion with breakthrough SARS-CoV-2 infection, sub-study 1	48/870 (5.5%)	65/889 (7.3%)	59/928 (6.4%)	124/1817 (6.8%)	1.34 (0.91 to 1.99)*	0.136	1.18 (0.80 to 1.76)*	0.402	1.27 (0.90 to 1.79)*	0.175
Proportion of participants with detectable anti-S IgGAM post vaccination, sub-study 2	514/536 (95.9%)	593/611 (97.1%)	617/631 (97.8%)	1219/1242 (98.1%)	1.32 (0.66 to 2.65)**	0.439	1.75 (0.85 to 3.60)**	0.129	1.49 (0.82 to 2.69)**	0.189

Abbreviations: IQR, interquartile range; OR, odds ratio. *Adjusted for age, sex, educational attainment, frontline worker status, number of people per bedroom, schoolchildren (5–15 years) at home with participant, primary vaccination course, previous SARS-CoV-2 infection, season of first vaccination, inter-dose interval, and use of anticholinergics. **Adjusted for age, sex, body-mass index, days from second vaccine dose to DBS sample, general health, season of second vaccination, inter-dose interval, primary vaccination course, and previous SARS-CoV-2 infection.

Table S7: Sensitivity analysis: continuous immunological outcomes in participants who did not report previous SARS-CoV-2 infection

	No offer	800 IU/day offer	3200 IU/day offer	Either offer	800 IU/day offer vs. no offer		3200 IU/day vs. no offer		Either offer vs. no offer	
					Adjusted % difference (95% CI)*	P	Adjusted % difference (95% CI)*	P	Adjusted % difference (95% CI)*	P
Anti-S IgGAM ratio (sub-study 2)	2.7 (1.9 to 3.8) [n=536]	2.8 (1.9 to 3.9) [n=611]	2.8 (1.9 to 4.0) [n=631]	2.8 (1.9 to 4.0) [n=1242]	-64.4% (-92.9 to 78.5)	0.204	-59.9% (-91.5 to 88.9)	0.242	-66.3% (-89.8 to 11.4)	0.074
S peptide-stimulated IFN-γ in whole blood supernatant, ng/mL (sub-study 3)	0.011 (0.003 to 0.025) [n=29]	0.013 (0.005 to 0.046) [n=28]	0.013 (0.005 to 0.024) [n=37]	0.013 (0.005 to 0.037) [n=65]	2.3% (-6.6 to 12.1)	0.616	-0.5% (-4.8 to 4.0)	0.823	1.7% (-4.0 to 7.6)	0.568

Data are median (IQR) [n] unless otherwise specified. Values below the limit of detection are presented as 0. *Adjusted for age, sex, ethnicity, body-mass index, pre-vaccination anti-S IgGAM, days from second vaccine dose to DBS sample, general health, inter-dose interval, and primary vaccination course.

Table S8: Exploratory responder analysis, comparing major immunological outcomes among participants randomised to either intervention arm who did vs. did not achieve end-trial 25(OH)D concentrations >75 nmol/L.

	**Participants achieving end-trial 25(OH)D ≥75 nmol/L**	**Participants achieving end-trial 25(OH)D <75 nmol/L**	**Adjusted percentage difference for ≥75 nmol/L *vs* <75 nmol/L***	**p value**
Post-vaccination anti-Spike IgGAM serology ratio	3.0 (2.1 to 4.1) [n=162]	2.5 (1.9 to 4.0) [n=45]	17.6% (-0.3 to 38.8)	0.054
Neutralising antibody titre	234.0 (76.3 to 492.3) [n=57]	241.1 (169.1 to 308.8) [n=9]	-26.2% (-67.9 to 69.4)	0.466
S peptide-stimulated IFN-γ in whole blood supernatant	0.013 (0.005 to 0.033) [n=56]	0.034 (0.007 to 0.049) [n=9]	-37.0% (-81.6 to 115.8)	0.455

Data are median (IQR) [n] unless otherwise specified. *Adjusted for age, sex, ethnicity, body-mass index, pre-vaccination anti-S IgGAM, days from second vaccine dose to DBS sample, general health, inter-dose interval, and primary vaccination course. Analysis restricted to participants randomised to either intervention arm, and classified according to total 25(OH)D values measured at study end, not by allocation.
